# Strategic Delay and Bargaining Over Public Insurance Coverage for Drugs in Australia

**DOI:** 10.1002/hec.70095

**Published:** 2026-03-12

**Authors:** Jing Jing Li, Anthony Harris

**Affiliations:** ^1^ Centre for Health Economics Monash Business School Monash University Melbourne Australia

**Keywords:** drug funding, dynamic bargaining, pharmaceuticals

## Abstract

Public funding for pharmaceuticals often follows repeated negotiation between manufacturers and a public agency, yet little empirical work examines how the timing of agreement reflects the economic structure of these interactions. Using a duration model of negotiations in Australia from 2005 to 2018, we assess whether observed patterns of delay and agreement align with dynamic bargaining theory under incomplete information. Agreements from 634 submissions for 400 therapies required a median of 16 months, and 71% of negotiation rounds ended without agreement. Therapies with lower expected value to the agency—reflected in higher incremental cost per QALY, greater budget impact, or evidence uncertainty—experienced longer delays and lower agreement rates, while those with strong clinical importance, perceived need, or elevated public interest were listed more quickly. Delays also varied across therapeutic classes and with a therapy's position in the sequencing of available treatments. The observed patterns point to a systematic listing rule in which therapies are funded when expected health gains justify their opportunity costs. They also support the view that the timing of agreement reflects strategic negotiation under uncertainty, not simply procedural delay.

## 1 Introduction

Bilateral bargaining between pairs of agents is pervasive in many economic environments. In the market for pharmaceuticals and other patented medical products, bargaining between third party payers and firms over insurance coverage and the associated per‐unit price or subsidy level is common. Yet empirical studies on the outcomes of bargaining remain limited. The general theoretical literature in economics has analyzed the equilibrium outcomes of bilateral bargaining in a variety of settings in what has been described as the “Nash program” (Binmore et al. 1986). Typically, the literature emphasizes equilibrium solutions, while empirical work examines how changes in market conditions or market structure affect those equilibria. Standard models focus on price in an agreement. In this paper, we focus instead on the time to agreement. We examine whether observed delays are consistent with a screening mechanism: high‐value therapies wait for the agency's valuation to converge with their bid, while low‐value therapies must concede early or face indefinite rejection. Using the empirical example of the Australian Pharmaceutical Benefits Scheme (PBS), we test some of the predictions of dynamic bargaining theory in the context of bilateral agreements (and disagreements) on subsidies to medicines between two players ‐ a public funding agency and a pharmaceutical company. We show that delay, agreement, and non‐agreement patterns in Australia's PBS reflect strategic negotiation under uncertainty, influenced by evolving evidence and fiscal constraints.

The last 50 years have seen a dramatic increase in the use, effectiveness, and cost of medicines. Concern about the affordability to consumers and to public insurers began in the 1990s and has been amplified by the rise of high‐priced biologic and targeted therapies in the 2000s. Governments and other third‐party payers have sought to reduce rising expenditure through a range of direct and indirect price and volume controls, including limits to insurance coverage and requirements to demonstrate value for money through health technology assessment (HTA) (Barnieh et al. 2014; Dylst et al. 2011; Lee et al. 2015; Towse et al. 2012). There has also been concern about the implications of public regulation of drug prices for the share of value going to each party and its effect on market dynamics, particularly on investment incentives for socially valuable innovation (Jena and Philipson 2008; Woods et al. 2024). The increased willingness of public health agencies and insurers to bargain over prices and coverage conditions for medicines has resulted in persistent criticism of the timeliness of decision‐making (Drummond and Sorenson 2009; Pearce 2012), with concerns that delays reduce not only the payoff to the pharmaceutical companies but also the net payoff to patients through lost therapeutic gains.

This paper develops an institutional bargaining framework in which the agency's latent threshold rules—such as benchmarks for an additional cost per QALY or budget impact—are embedded in a dynamic negotiation process. By treating the timing of agreement as the equilibrium outcome, the framework highlights how these implicit thresholds and institutional dynamics influence delay and the rate of agreement in the Australian drug subsidy process. We examine how these features are revealed in the conditions under which agreements are reached, how long negotiations last, how delay affects the likelihood of agreement, and how interdependencies across therapeutic classes and indications shape bargaining outcomes. We also consider whether sequencing strategies for multi‐therapy drugs and international reference pricing constraints influence the evolution of offers and the probability of agreement. Our empirical analysis links these structural elements to observable behavior using a duration model of time to agreement and within‐therapy models of belief updating. The findings provide empirical evidence consistent with dynamic bargaining models in which the timing of agreement is an endogenous feature of negotiation, and not merely the consequence of a bureaucratic process.

We examine:

- Which therapies reach agreement and which do not,
- How long negotiations last and how duration varies across therapies and classes,
- How delay affects the probability of eventual agreement,
- How therapeutic alternatives and class saturation shape bargaining outcomes,
- How public interest shifts the timing or likelihood of agreement, and
- Whether earlier agreements for multi‐therapy drugs influence later indications.

Using data from 634 negotiation rounds for 400 therapies, we find that therapies with higher incremental cost per QALY, greater budget impact, or more uncertain evidence experience longer delays and lower agreement rates, while those with strong clinical importance or elevated public interest reach agreement more quickly. Delays also vary across therapeutic classes and with a therapy's position in the sequencing of available treatments. These empirical patterns are consistent with dynamic bargaining models in which delay reflects strategic behavior under uncertainty rather than purely administrative factors.

Classic dynamic bargaining models (Cramton 1992; Fudenberg et al. 1985, 1987) explicitly model delay and timing, but under standard assumptions, their equilibria often collapse to prices that reflect static surplus division ‐ that is, the distribution of the net gains from bargaining. In contrast, applied Nash‐in‐Nash frameworks (e.g., (Collard‐Wexler et al. 2019; Ho and Lee 2017, 2019)) that emphasize bargaining interdependence within networks adopt this static representation directly and model surplus division. Our framework builds on these contributions but considers dynamic strategic behavior and treats the timing of agreement itself as the equilibrium outcome, consistent with the institutional reality of repeated submissions and latent threshold rules in the Australian drug funding decision process.

The paper is organized as follows. Section 2 reviews some of the applications of bilateral bargaining models in economics and health. Section 3 describes the theoretical framework and the empirical models. Section 4 describes the data used. Section 5 examines whether observed listing patterns in the Australian pharmaceutical market are consistent with the implications of bargaining models. Section 6 describes the results, which are discussed in Section 7 with conclusions in Section 8. Supplementary appendices provide a more extensive theoretical framework (Appendix 1) and robustness checks of the empirical estimates (Appendices 2, 3 and 4).

## 2 Related Literature

Empirical work on bilateral bargaining over health technology pricing has focused mainly on the effects of competition and price discrimination. For example, Grennan (2013) shows the importance of bargaining power in determining the final price and the power of a single purchaser to counteract the effect of lower competition on prices. In the pharmaceutical market, there have been several game‐theoretic studies of pricing and access policies, such as reference pricing and performance‐based risk‐sharing arrangements (Antonanzas et al. 2011; Barros 2011; Critchley and Zaric 2019), and the impact of a drug manufacturer's marketing decision on coverage, pricing, and social welfare (Wright 2004). Recent work embeds bilateral negotiations within broader strategic contexts. Ho and Lee (Ho and Lee 2017, 2019) apply a Nash‐in‐Nash framework to hospital–insurer bargaining, showing how exclusion of hospitals, falling marginal value from enlarging a network, and budget pressure determine equilibrium outcomes. In terms of comparable non‐health related empirical dynamic bargaining models with one‐sided asymmetric information, Ambrus et al. (2011), in the very different setting of historical ransom negotiations and Backus et al. (2020) in online auctions find results consistent with incomplete information models. They document common sequences of failure to reach agreement and show that bargaining power and better outside options improve agents' outcomes.

A related empirical literature has examined the revealed public preferences for the outcomes of pharmaceutical funding. In an analysis of the UK National Health Service, Dakin et al. (2015) found that the additional monetary cost per unit of health gain alone predicted 82% of the decisions to fund drugs, while (Harris et al. 2008; Harris et al. 2016) concluded that cost‐effectiveness, clinical efficacy, cost to government and disease severity were significant factors in drug funding decisions in Australia. These analyses considered the likelihood of a subsidy given the expected net benefits of the treatment, but they did not consider the strategic interaction of the parties nor the dynamics of the negotiation.

We build on models of bilateral bargaining, including signaling models and the Nash‐in‐Nash framework, and extend them to a setting where negotiations are repeated, information evolves over time, and decisions depend on the broader context of other therapies already funded or under consideration. Using data from the Australian PBS, we estimate a reduced‐form hazard model of time to agreement, linking empirical patterns to structural features of dynamic negotiation, and examine whether delay and disagreement are consistent with incomplete information, strategic signaling, and interdependent listing conditions. Unlike typical empirical bargaining studies that focus on pricing as outcomes, here the timing and likelihood of agreement are the outcomes of bargaining. Our main contribution then is to link dynamic bargaining theory to empirical duration modeling of funding decisions, treating delay as an endogenous outcome rather than noise.

## 3 Theoretical Framework

### 3.1 A Dynamic Threshold Rule

Funding agencies that use HTA are often described as applying an implicit threshold when deciding whether to fund a therapy. In the empirical literature, this threshold is typically inferred from static logit or probit models of listing decisions on ICERs, clinical importance, disease severity, and budget impact (Dakin et al. 2015; Harris et al. 2008; Harris et al. 2016). These approaches treat decisions as single events and interpret coefficients as revealing a fixed willingness‐to‐pay threshold. However, they abstract from the fact that negotiations unfold over time, evidence accumulates, and the set of therapies already subsidized on the national formulary changes, altering the resources and therapeutic class‐level constraints faced by the agency.

We formalize the negotiation as a dynamic game embedded within a Nash‐in‐Nash environment. The agency's listing decision at time t depends on two conditions: whether the therapy's assessed value is sufficiently high relative to its (QALY‐adjusted) price, and whether the expected expenditure fits within the remaining budget. Let $\psi_{it}$ denote the agency's latent valuation of therapy i, $p_{it}$ its proposed price, and $b_{it}$ the expected expenditure. The agency accepts if:

$$\psi_{it}-\frac{p_{it}}{\lambda }\geq \delta_{it}\text{and}\frac{b_{it}}{\lambda }\leq B_{t}^{\text{resid}}$$

Here, $\psi_{it}$ captures the value of expected health gains, clinical importance, uncertainty, and public interest; λ is the system‐wide shadow price of a QALY; and $p_{it}/\lambda$ converts price into QALY‐equivalent units. For tractability, we treat the shadow price of a QALY λ as time‐invariant over the study period, with temporal variation in opportunity cost absorbed into the agency's latent valuation $\psi_{it}$ and budget feasibility term. The term $\delta_{it}$ is a saturation threshold: the minimum margin by which valuation must exceed adjusted price to justify listing another therapy in the same class, rising as the class becomes more crowded. $B_{t}^{\text{resid}}$ denotes the agency's residual budget capacity at time t, after accounting for existing commitments, new listings must satisfy the feasibility condition $b_{it}/\lambda \leq B_{t}^{\text{resid}}$.

This representation provides a transparent, static acceptance rule that can evolve over time as valuations, evidence, and class‐level conditions change.

### 3.2 Dynamic Bargaining Under Incomplete Information

We embed this threshold rule in a dynamic bargaining environment. Each negotiation occurs within a broader portfolio of therapies, where cumulative approvals affect budget capacity and the marginal value of listing additional drugs. Companies anticipate these constraints and time submissions strategically—for example, by submitting high‐value indications first to anchor prices or by delaying until stronger evidence becomes available.

A company's timing strategy balances the immediate gains from listing against the value of waiting. Delay is therefore not merely frictional but arises from two strategic sources:

1. Signaling under asymmetric information: High‐value drugs can afford to delay agreement to signal quality, separating themselves from low‐value drugs that cannot bear the cost of delay and concede earlier (Cramton 1992; Fudenberg and Tirole 1986, 1991). In our context, this might take the form of reputational signaling within a drug (early experience in one indication increases value for later therapies in other indications).
2. Value of waiting. Delay also arises because firms anticipate that the negotiation environment may improve over time. Evidence may strengthen (raising $\psi_{it}$), scrutiny within therapeutic class may ease (lowering $\delta_{it}$), or budget capacity may improve, making future rounds more favorable. When the expected payoff from all future negotiation opportunities if the firm does not agree today (the *continuation value*) exceeds the payoff from immediate agreement, the option value is positive and the company delays (Dixit and Pindyck 1994).

### 3.3 Interdependencies Across Therapies

Negotiations occur within therapeutic classes, each with its own history of approvals, cumulative expenditure, and saturation dynamics. Such interdependencies are central to Nash‐in‐Nash bargaining models, where each bilateral negotiation is influenced by the broader portfolio of agreements (Collard‐Wexler et al. 2019; Gowrisankaran et al. 2015; Ho and Lee 2017, 2019). When several therapies compete for space in a therapeutic class, the marginal value of adding one more declines, raising the effective threshold $\delta_{it}$. Companies anticipate these class‐level dynamics when choosing when to submit, how to price, and how to adjust evidence packages.

Our framework, therefore, extends static revealed‐threshold approaches by allowing the threshold to evolve with negotiation dynamics, evidence, and class‐level conditions.

### 3.4 Sequencing Strategies for Multi‐Therapy Drugs

For drugs with multiple indications, sequencing becomes an additional strategic lever. A strong initial submission can build credibility and increase the likelihood that later indications are accepted, while subsequent submissions for close substitutes may face reduced marginal value if the therapeutic class has many close substitutes (*saturated*) or the agency faces greater fiscal and political risk in expanding coverage.

In the dynamic threshold framework, sequencing is therefore one component of the company's timing strategy: firms choose the order of submissions to influence the agency's evolving valuation $\psi_{it}$, manage saturation $\delta_{it}$, and influence the continuation value of future indications.

### 3.5 International Reference Pricing as a Continuation‐Value Constraint

International reference pricing further conditions the company's strategy. A firm that lowers its price in Australia risks triggering lower reference prices in other markets, reducing global revenues. This acts as a continuation constraint: firms cannot simply resubmit at a lower price after rejection without affecting their global position. As a result, some therapies are delayed or withdrawn because outside‐option constraints prevent feasible price reductions. In Nash‐in‐Nash terms, international reference pricing raises the firm's disagreement payoff, shifting the feasible bargaining set and making delay or withdrawal a rational equilibrium outcome.

#### 3.5.1 Empirical Mapping

Our primary empirical analysis translates this structure into a reduced‐form hazard model of time to agreement. Each predictor corresponds directly to a structural element of the bargaining framework (see Table 1). Incremental cost‐effectiveness ratios capture expected net payoff; confidence in the evidence reflects information revelation; therapeutic saturation represents interdependence across negotiations; public interest shifts the agency's valuation; and budget thresholds embody feasibility constraints within the agency's portfolio.

**TABLE 1:** Variables and key concepts.

Construct	Variable	Definition/Coding	Source
Expected net value	ICER band (1–6)	Incremental cost‐effectiveness ratio reported in six PBAC bands. Coded 1 = < $15k/QALY to 6 = > $200k/QALY. Higher values indicate lower net value to the agency.	PSD
	Budget impact > $10m	Indicator = 1 if projected annual PBS expenditure exceeds $10 million Australian dollars. Threshold reflects the long‐standing PBAC category for high‐cost therapies.	PSD
	Clinical uncertainty	1 = PBAC judged the clinical evidence to have *considerable* uncertainty; 0 = *no or limited* uncertainty.	PSD
	Economic uncertainty	1 = PBAC judged the economic model to have *considerable* uncertainty; 0 = *no or limited* uncertainty.	PSD
	Clinically important effect	1 = PBAC judged the treatment effect to be clinically important; 0 = otherwise.	PSD
	FDA priority review	1 = therapy received FDA priority review designation; proxy for severity/need/clinical urgency.	FDA (2025)
Therapeutic class saturation	ATC‐4 saturation	Number of other drugs listed in the same ATC level‐4 class at the time of submission. Captures within‐class crowding and declining marginal value of additional therapies.	ATC ((WHOCC 2024)
Public interest	Google Trends (standardized)	Average weekly difference between the drug‐specific Google Trends index and the overall Health‐category index over the 12 months prior to the PBAC meeting. Captures drug‐specific search intensity relative to general health‐related search activity. Standardized (z‐score) for regression.	Google Trends (2024)
International reference pricing	US VA price	U.S. Veterans Affairs Federal Supply Schedule price at submission date. Used only in mediation analysis (not included in duration models).	US VA FSS (2024)
Negotiation dynamics	Round number	Submission round within a therapy (1–6). Used in within‐therapy fixed‐effects models to capture belief updating and concessions.	PSD

Abbreviations: ATC=World Health Organisation Anatomical Therapeutic Chemical Classification; FDA = The U.S. Food and Drug Administration; PBAC=Pharmaceutical Benefits Advisory Committee; PBS=Pharmaceutical Benefits Scheme; PSD = public summary document, which is a public meeting documentation of the PBAC; U.S. VA FSS=United States Veteran Affairs Federal Supply Schedule.

This structural–empirical mapping allows us to assess whether observed patterns of delay and agreement are consistent with the constraints and incentives implied by dynamic bargaining under uncertainty. We also exploit the panel structure of the data to estimate linear fixed‐effects models across therapies and negotiation rounds. These models identify within‐therapy dynamics in agency confidence, projected budget impact, and public interest. For multi‐therapy drugs they allow us to examine whether prior agreement in one indication alters the likelihood of agreement in subsequent indications, consistent with sequencing strategies and evolving continuation values.

## 4 Empirical Strategy

We develop an empirical strategy that captures how negotiations unfold over time. The agency's acceptance rule implies that agreement depends on whether a therapy's assessed value exceeds its adjusted price by a sufficient margin and whether the expected expenditure fits within the remaining budget. These conditions motivate a reduced‐form hazard model of time to agreement, with predictors chosen to reflect the key elements of the bargaining environment: valuation relative to price (ICER), budget pressure (projected expenditure), evidence quality (clinical and economic credibility), therapeutic class saturation (capturing both declining marginal value as classes become crowded and the increasing budget exposure from adding further similar therapies), continuation‐value dynamics (how evolving evidence and class conditions affect the value of waiting), and public interest (which may shift the agency's valuation or salience). Although the underlying decision problem is dynamic, the agency's valuation path is unobserved, so our empirical strategy focuses on the static implications the dynamic model yields for delay and the observable adjustments that occur across negotiation rounds.

We use two complementary empirical approaches to characterize negotiation dynamics and the determinants of agreement. The first is a time‐to‐event framework, modeling the duration from the first submission to the PBAC to the first agreement to list a therapy at the proposed price and population. This approach incorporates both completed agreements and censored observations, recognizing that many therapies do not reach agreement within the observation window. We estimate Cox models with a common baseline hazard and then stratify by ATC level‐2 to allow baseline hazards to vary across therapeutic areas. This captures the expectation that delay dynamics differ across broad areas with distinct expenditure profiles, saturation patterns, and continuation values, while preserving common effects of ICER, uncertainty, budget thresholds, and public interest.

To aid interpretation, we compute predicted marginal median times to agreement for each covariate using the Cox model with a base set of values for other predictors. These margins provide an intuitive measure of how observable characteristics shift expected negotiation duration. The Cox model does not require parameterization of the baseline hazard and assumes that the relative rate of agreement between therapies is constant over time. We assess this assumption through robustness checks using parametric survival models and a logit specification. We also report results of tests of the proportional‐hazards assumption using Schoenfeld residuals (Appendix 4, Table A.4.1). Therapies are followed from the date of their first proposal until agreement or censoring at the end of the observation period, with the Efron approximation used to adjust for tied survival times.

We tested interactions among covariates, but none were significant; the final model, therefore, includes only main effects. Hazard ratios and 95% confidence intervals clustered by therapy are reported, and model fit is assessed using Cox–Snell residuals. Delayed entry is applied for therapies with proposals predating 1 March 2005. In the primary analysis, therapies not listed within 4 years of the first proposal are censored to avoid undue influence from extreme durations; results using the full dataset and censoring at 5 years are also reported. Table 1 maps each empirical variable to its corresponding structural construct.

The second empirical approach examines how key components of the agency's valuation evolve within a therapy across successive negotiation rounds. Because negotiation dynamics, belief updating, and concessions occur within a therapy rather than across drugs, we estimate linear fixed‐effects models at the therapy level. In these models, the ICER is treated as a six‐level ordered variable coded 1–6, corresponding to the six ICER bands reported in PBAC documents. We treat this scale as approximately continuous for the purposes of the linear fixed‐effects specification to capture within‐therapy changes in the ICER band across negotiation rounds while controlling for all time‐invariant characteristics of the therapy. Because the ICER bands are ordinal rather than cardinal, we complement the linear fixed‐effects model with an ordered logit specification to confirm that the direction and significance of effects are robust to functional‐form assumptions. These models identify round‐by‐round changes in the accepted ICER band, the agency's confidence in the evidence, and the projected budget impact, net of all time‐invariant characteristics of the therapy, such as underlying clinical value, indication size, or baseline cost structure. This specification aligns directly with the theoretical framework, in which the agency updates beliefs about a specific therapy as negotiations proceed and the company adjusts its offer or supporting evidence.

For each outcome—ICER band, confidence index, and the binary indicator for projected annual spending above $10 million—we estimate pooled linear models and therapy fixed‐effects models, with complementary ordered logit or random‐effects logit specifications to account for the ordinal or binary nature of the outcomes. These additional models confirm that the direction and magnitude of the within‐therapy effects are not sensitive to functional form.

Sequencing across indications of the same drug operates at the drug level; because it does not vary within a therapy, it cannot be identified in therapy fixed‐effects models. We therefore estimate the hazard function within drug strata, which removes between‐drug heterogeneity and allows us to examine whether cross‐therapy spillovers are present. We complement this with linear drug fixed‐ and random‐effects models of the probability of agreement. For drugs with multiple indications, we assess whether early agreement in one indication is associated with higher acceptance probabilities for subsequent indications. Such patterns are consistent with mechanisms emphasized in both screening models—where early success reduces uncertainty about underlying value—and dynamic bargaining frameworks in which prior agreements shift beliefs, credibility, or the marginal value of later indications. We do not attempt to distinguish these mechanisms structurally, but the empirical associations provide evidence on whether sequencing effects persist after accounting for drug‐level heterogeneity.

We anticipate that the main effect of a high international price is to increase the bid price for the therapy and increase the ICER. We therefore exclude overseas prices in the duration analysis as their primary effect operates through the ICER rather than directly on the hazard of agreement. We use structural equation modeling to test whether the U.S. price indirectly affected the probability of an agreement through mediation of the value of the offer to the payer.

All analyses were conducted using STATA version 17.0 (StataCorp LP, College Station, TX, U.S.). We report 95% confidence intervals and test the significance of covariates in all analyses at the 1%, 5% and 10% levels. This empirical structure provides a direct mapping from theory to observable behavior, allowing us to test whether patterns of delay and agreement are consistent with dynamic bargaining under uncertainty. The next section describes the data used to operationalize these constructs.

## 5 Data

Our analysis uses detailed data from Australia's national public pharmaceutical insurance scheme, the Pharmaceutical Benefits Scheme (PBS), which provides a transparent record of negotiation rounds, evidence assessments, and outcomes. The structure of the PBS process—fixed submission cycles, documented committee deliberations, and frequent resubmissions—allows us to observe how negotiations evolve within and across therapies. The PBS was the first national system to require formal economic evidence for subsidy decisions, and its long‐running, highly structured negotiation process—three fixed meetings each year,^1^ detailed documentation of offers and outcomes, and a long history of iterative submissions—provides an unusually rich setting for studying bargaining dynamics. Companies submit proposals to list a therapy at a specified price for a given indication, and the Pharmaceutical Benefits Advisory Committee (PBAC), a semi‐independent agency, evaluates the evidence and recommends whether the therapy should be funded. Accepted therapies are subsidized with a low, fixed patient contribution; rejected therapies may be resubmitted, and resubmissions are common. Although firms can technically reapply at the next meeting, preparation requirements mean that most resubmissions occur after an additional cycle. The evaluation process itself takes 17 weeks, during which firms may address concerns raised by the committee.

PBAC recommendations are based on expected expenditure, treatment effectiveness, cost‐effectiveness relative to current therapy, and the committee's confidence in the supporting evidence. They also consider less quantifiable factors such as clinical need, severity of illness, equity, and the availability of alternatives. Together, these elements form the agency's assessment of the therapy's value relative to its cost.

**FIGURE 1:**
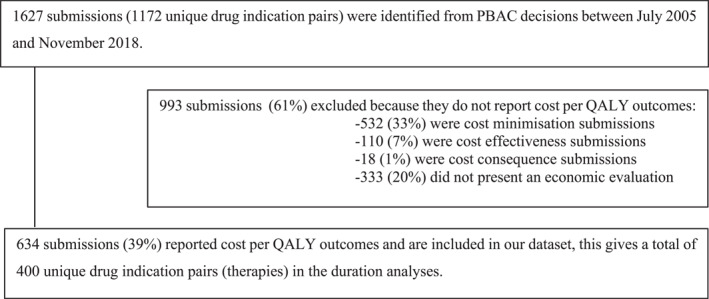
Flow Diagram of submissions (i.e., rounds of negotiations) included in the analyses.

Our primary data source is the set of public summary documents (PSDs) published from July 2005 onwards (PBAC Decisions 2024). From all submissions between July 2005 and November 2018 (*n* = 1627), we extracted those that reported QALY‐based evidence and involved a negotiated price (*n* = 634). We excluded cost‐minimisation submissions and those without a comparable outcome measure, as these do not permit meaningful comparisons of value across therapies. Submissions were grouped into drug–indication pairs (therapies), with each submission representing a negotiation round. The final dataset contains 634 rounds across 400 therapies and 270 drugs, with up to six rounds per therapy (Figure 1).

The main outcome in the duration analysis is the number of years from the first submission until the PBAC recommends funding at the proposed price. A recommendation at the requested price is coded as agreement; all other outcomes—conditional recommendations, rejections, or deferrals—are treated as non‐agreement.^2^

To characterize the agency's assessment of value, we use the incremental cost‐effectiveness ratio (ICER), reported in six bands, where higher ICERs imply lower value relative to current therapy. We proxy for budget pressure using an indicator of whether projected annual expenditure exceeds AUD 10 million, a level that typically attracts heightened scrutiny. The committee's confidence in the evidence is captured through three indicators reflecting whether the PBAC judged the clinical effect to be meaningful, the clinical evidence to be reliable, and the economic model to be credible. Because the PBAC does not value all QALY gains equally and documentation on severity and need is inconsistent, we proxy patient need using the U.S. FDA's priority‐review designation (FDA 2025).

Therapeutic class structure is captured using the number of drugs listed at ATC level 4 at the time of negotiation, reflecting the degree of saturation within narrowly defined clinical subgroups where marginal value declines as more similar therapies are listed (e.g., L01XC monoclonal antibodies for cancer). Broader continuation‐value dynamics are stratified using ATC level 2 categories, which correspond to therapeutic areas (e.g., L01 cancer drugs or A10 diabetes treatment drugs) with distinct budget pressures and evidence expectations (WHOCC 2024).

Public interest is proxied using a standardized Google Search intensity index (Google Trends 2024). For each therapy, we construct a weekly series of drug‐specific search volume over the year prior to submission. We express each week's value as the percentage change relative to the therapy's first observed week, subtract the contemporaneous change in the overall Google “Health” category to remove platform‐wide movements, and then standardize the resulting series. This places all therapies on a common scale and allows the hazard coefficient to be interpreted as the effect of a one‐standard‐deviation increase in therapy‐specific public interest. This measure captures shifts in public attention specific to the therapy that may influence the agency's valuation or the political salience of the submission.

For analyses involving global pricing constraints, we use U.S. prescription drug prices from the Veterans Affairs Federal Supply Schedule at each proposal date (US VA FSS, 2024).

All PSD data were double‐extracted by experienced evaluators using a prespecified coding manual. Evidence‐quality ratings were coded blind to final decisions, and discrepancies were resolved by consensus, using objective statements in the PBAC documentation. These data allow us to construct the variables required for the empirical strategy outlined in Section 4 and to track negotiation dynamics over time. We now turn to the results.

## 6 Results

### 6.1 Descriptive Patterns

**FIGURE 2:**
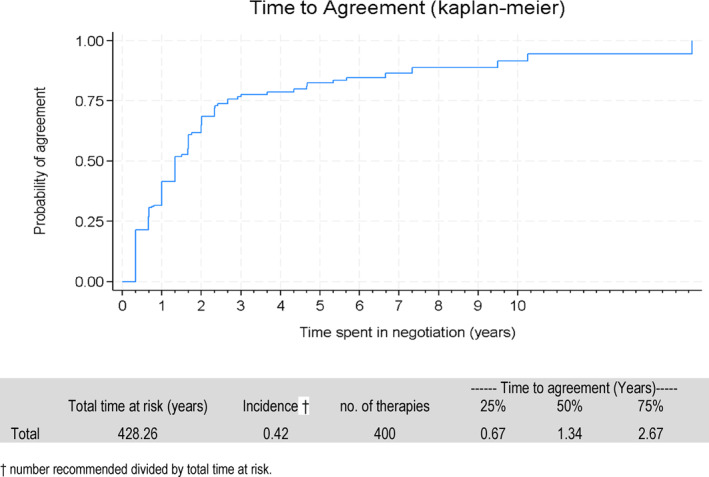
Kaplan‐Meier curve of time to agreement from date of first proposal to the agency.

Table 2 summarizes the characteristics of the 634 submissions covering 400 distinct therapies. Forty‐five percent of therapies eventually reached agreement, and the median time to agreement was 1.34 years (IQR: 0.67–2.67), as shown in Figure 2. Therapies underwent an average of 1.5 negotiation rounds, with a maximum of six. Seventy‐one percent of negotiation rounds did not result in agreement, reflecting the iterative nature of PBS submissions.

**TABLE 2:** Descriptive patterns.

Characteristics	*n* (%) of submissions	*n* (%) of therapies^a^
Number of negotiations (i.e. number of cost per QALY submissions)	634	—
Total number of drug/indication pairs (therapies)	400	400
Number of rounds of negotiations per therapy, mean (sd) median (range)	1.51 (0.78) 1 (1,6)	—
Number that reached agreement^c^	181/634 (29%)	181/400 (45%)
FDA priority need	158/634 (25%)	111/400 (28%)
# other drugs in ATC level‐4, mean (SD)	3.5 (1.8)	—
Evidence from randomized trials	562/634 (89%)	358/400 (90%)
Clinically important difference between drug and comparator	363/634 (57%)	261/400 (65%)
Considerable clinical uncertainty	286/634 (45%)	208/400 (52%)
Considerable economic uncertainty	432/634 (68%)	307/400 (76%)
% change in Google Trends in the year leading up to meeting		
Mean (SD)	7% (55%)	—
Median (range)	−1% (−99%, 332%)	—
Less than 0%	318 (50%)	242 (61%)^b^
Between 0% and < 50% increase	237 (37%)	196 (49%)^b^
50% increase to < 100% increase	44 (7%)	44 (11%)^b^
100% increase to < 150% increase	19 (3%)	19 (5%)^b^
Greater than 150% increase	16 (3%)	16 (4%)^b^
ICER ($/QALY gained) band in PSD		—
Less than $15,000	81 (13%)	70 (18%)^b^
$15,000 to $45,000	191 (30%)	155 (39%)^b^
$45,000 to $75,000	215 (34%)	158 (39%)^b^
$75,000 to $104,000	61 (10%)	54 (14%)^b^
$105,000 to $200,000	48 (8%)	45 (11%)^b^
Greater than $200,000	38 (6%)	30 (8%)^b^
Budget impact > $10Million (AUD) per annum	386/634 (61%)	244/400 (61%)

Abbreviations: # = number, ICER = incremental cost‐effectiveness ratio, IQR = interquartile range, PSD = public summary document, SD = standard deviation.

^a^ Therapies are unique drug/indication pairs; submissions for the same drug for distinctly different indications were considered to be separate therapies in the analyses.

^b^ Does not add up to 100% due to the values changing between successive submissions.

^c^ Agreement is reached when the PBAC recommends listing at the price and population proposed without further restrictions.

FDA priority designation applied to 28% of therapies, and 61% were projected to exceed the $10 million annual budget threshold. Considerable clinical uncertainty was recorded in 52% of therapies and economic uncertainty in 76%. Therapeutic class saturation—measured as the number of other therapies listed in the same ATC level‐4 class—averaged 3.5. Public interest, measured using Google Trends, showed substantial variation, with a mean change of 7% and a wide range from −99% to +332%.

### 6.2 Determinants of Time to Agreement

**FIGURE 3:**
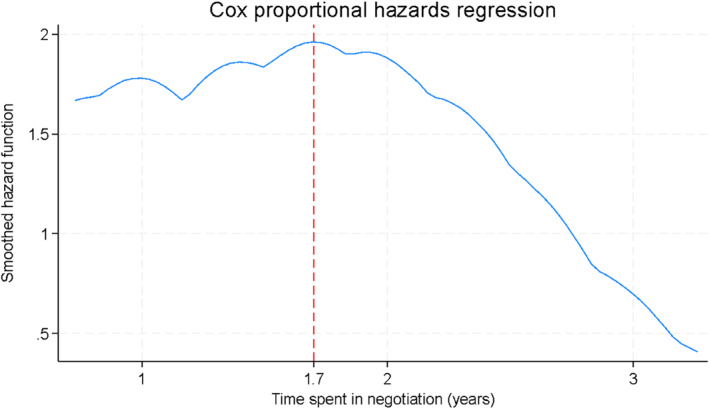
Baseline hazard for reaching an agreement over time.

Table 3 reports the Cox proportional hazards estimates, with associated baseline hazards for reaching an agreement over time shown in Figure 3. Higher ICER categories were associated with progressively lower hazards of agreement. Relative to therapies with ICER < $15,000/QALY, the hazard ratio was 0.55 (95% CI: 0.36–0.84) for ICER $45,000–$75,000 and 0.15 (95% CI: 0.04–0.48) for ICER > $200,000. Considerable clinical uncertainty (HR = 0.50; 95% CI: 0.33–0.75) and economic uncertainty (HR = 0.36; 95% CI: 0.26–0.51) were also associated with substantially lower hazards of agreement.

**TABLE 3:** Cox model results.

Variables	HR (95% CI)^a^	
	Cox model	Cox therapeutic area strata^b^
FDA priority need	1.45** (1.03, 2.04)	1.09 (0.72,1.65)
Number of drugs in ATC level‐4	0.95 (0.88, 1.03)	0.93 (0.84, 1.03)
Clinical uncertainty	0.50*** (0.33, 0.75)	0.58** (0.38,0.88)
Clinically important difference	1.55** (1.07, 2.24)	1.37 (0.92, 2.05)
Economic uncertainty	0.36*** (0.26, 0.51)	0.31***(0.22–0.45)
ICER band (comparisons vs. ICER < $15,000)		
$15,000–$45,000	0.67* (0.43, 1.04)	0.62**(0.38, 0.99)
$45,000–$75,000	0.55*** (0.36, 0.84)	0.57*(0.32, 1.01)
$75,000–$105,000	0.47** (0.23, 0.95)	0.46*(0.21, 1.00)
$105,000–$200,000	0.22** (0.07, 0.71)	0.27**(0.08, 0.85)
$200,000	0.15*** (0.04, 0.48)	0.08**(0.01, 0.75)
Budget impact > $10million per year	0.77 (0.57, 1.05)	0.69**(0.49, 0.96)
Google Trends (each SD from mean)	1.24*** (1.10, 1.39)	1.19***(1.05, 1.35)
Number of submissions	625	625

*Note:* Confidence intervals adjusted for therapy clusters.

^a^ Censored at 4 years plus delayed entry (1 March 2005 when PSDs became publicly available).

^b^ Stratified on ATC level‐2 classification.

^*^ *p* ≤ 0.1.

^**^ *p* ≤ 0.05.

^***^ *p* ≤ 0.01.

Therapies judged to have a clinically important effect had higher hazards of agreement (HR = 1.55; 95% CI: 1.07–2.24). FDA priority designation was similarly associated with faster agreement (HR = 1.45; 95% CI: 1.03–2.04). Public interest was positively associated with agreement: a one‐SD increase in Google Trends was associated with a hazard ratio of 1.24 (95% CI: 1.10–1.39). Budget impact showed a negative association with agreement. Therapies forecast to exceed $10 million per year had a hazard ratio of 0.77 (95% CI: 0.57–1.05). When baseline hazards were stratified by ATC level 2, the effect became statistically significant (HR = 0.69; 95% CI: 0.50–0.95). Therapeutic saturation was negatively signed but not statistically significant in either specification. This aligns with Appendix 2, Table A.2.1, where saturation coefficients are consistently negative across Cox and parametric models but remain imprecise.

Predicted margins in Table 4 illustrate the magnitude of these effects. For example, considerable economic uncertainty increased the median predicted time to agreement by 16 months, while FDA priority designation and a 1 standard deviation increase in the Google search index each reduced it by 4 months.

**TABLE 4:** Predicted margins for time to agreement.

	Difference in time to agreement from base case prediction of 1 year^a^		
Variables	Median	25%	75%
FDA priority = 1	−0.33	0.00	−0.34
Number of drugs in ATC level‐4 (1 SD from the mean)	0.00	0.01	0.33
Clinical uncertainty	0.67	0.42	NE
Clinically important difference	−0.33	0.00	−0.34
Economic uncertainty	1.34	0.67	NE
ICER band = 1 < $15K	−0.66	0.00	−0.67
ICER band = 2 $15–45K	−0.33	0.01	−0.17
ICER band = 3 $45–75K	—	—	—
ICER band = 4 $75,000 ‐ $105,000	0.00	0.01	0.33
ICER band = 5 $105,000 ‐ $200,000	1.00	0.67	NE
ICER band = 6 > $200,000	NE	1.01	NE
Budget impact > $10 Million per annum	0.33	0.01	0.66
Google Trends (SD from the mean)	−0.33	0.00	−0.33

^a^ base scenario where ICER band = 3 (< $45,000‐$75,000), all other categories set to zero and continuous variables set at the mean. The median time to agreement is 1 year (IQR: 0.33,1,67) NE = not estimable.

### 6.3 International Reference Pricing

We also examined whether international prices spill over into Australian negotiations. Using a mediation analysis of U.S. prices, we find a small but statistically significant indirect effect operating through the ICER: higher U.S. prices slightly worsen the ICER band and thereby reduce the probability of agreement. The direct effect of U.S. prices on agreement is close to zero and not statistically significant, consistent with the agency not explicitly referencing overseas prices in its decisions (full details in Appendix 3).

### 6.4 Within‐Therapy Dynamics

Table 5 presents the fixed‐effects regressions examining changes across negotiation rounds. Agency confidence increased with each additional round: relative to the first submission, confidence rose by 0.42 points in round 2 (95% CI: 0.25–0.58) and by 0.82 points in round 3 (95% CI: 0.58–1.07). The ICER band (coded 1–6) declined across rounds, indicating movement into more favorable cost‐effectiveness categories. Although the ICER band is ordinal, we treat it as approximately continuous in the linear fixed‐effects model to capture within‐therapy changes. Projected budget impact showed no consistent within‐therapy pattern. Appendix 3 Tables A.3.1‐A.3.3 show that these results are robust to alternative model specifications.

**TABLE 5:** Linear therapy fixed effect regressions of agency's confidence in value, ICER, and budget impact on prior agreement and negotiation rounds.

	Confidence	ICER	Budget impact
	(1)	(2)	(3)
Prior drug agreement	−0.84** (−1.66, −0.02)	−0.80 (−2.11, 0.51)	−0.05 (−0.07, 0.18)
Prior negotiation = 1	0.42***(0.25, 0.58)	−0.222* (−0.43, 0.01)	−0.06 (−0.14, 0.02)
Prior negotiation = 2	0.82***(0.58, 1.07)	−0.55*** (−0.83, −0.26)	−0.07 (−0.22, 0.07)
Prior negotiation = 3	0.95 (0.61, 1.28)	−0.41 (−1.04, 0.22)	−0.07 (−0.31, 0.16)

*Note:* Robust or bootstrap confidence intervals *n* = 634. Col 1 Linear, therapy fixed effect regression of index (1–4) of greater confidence in the evidence where the estimated parameters are the change in the index for a prior agreement for the drug or over subsequent negotiation rounds for the drug after the first. Controls included for FDA priority, Google Trends (SD above the mean), budget impact, and the number of potential substitutes. Col 2 Linear, therapy fixed effect regression of ICER index (low = 0 to high = 6) for a prior agreement for the drug or over subsequent negotiation rounds for the therapy controls for FDA priority, Google Trends (SD above the mean), quality of evidence, the number of potential substitutes, and US drug price. Col 3 Linear, therapy fixed effect regression of binary indicator if the cost to government is expected to be over $10m where the estimated parameters are the increased odds of the cost of government being over $10m for a prior agreement for the drug or over subsequent negotiation round for the therapy after the first round. Controls for FDA priority, Google Trends (SD above the mean), quality of evidence, and the number of potential substitutes.

^*^ *p* ≤ 0.1.

^**^ *p* ≤ 0.05.

^***^ *p* ≤ 0.01.

### 6.5 Sequencing in Multi‐Indication Drugs

Sequencing effects differed across model specifications. Cox models stratified by drug and the linear fixed‐effects model show negative but imprecise effects of prior listing on subsequent agreement (Table 6, cols. 1, 3). In contrast, random‐effects models grouped at the drug level (Table 6, cols. 2, 4) showed positive effects. The potential inferences from these contrasting results are discussed in Appendix 3, but none of the sequencing estimates are statistically significant at the 5% level in any specification. The number of drugs with multiple indications was limited, and indications varied substantially in scope, constraining the precision of these estimates.

**TABLE 6:** Regression for the effect of prior agreement for the drug on the rate of subsequent agreement for a therapy, conditional on observed characteristics of therapy at the time of negotiation and unobserved heterogeneity associated with the drug, modeled as fixed (strata) [FE] or random effects [RE].

	Cox (drug strata)	Logit RE	Linear FE	Linear RE
	(1)	(2)	(3)	(4)
	HR	OR	β	β
Prior drug agreement	0.52*	1.52	−0.05	0.07
95% CI	(0.24–1.13)	(0.87–2.66)	(−0.17 0.07)	(−0.01 0.15)
Observations	625	634	634	634
Number of groups	270	270	270	270

*Note:* All regressions control for the variables included in the main analysis of time to listing in Table 3. Col 1 reports hazard ratio from Cox regression stratified by drug. Col 2 reports the odds ratio from a logistic regression with a drug random effect, and Col 3 reports the coefficient from a linear regression with a drug fixed effect. Col 4 reports the coefficient from a linear regression with a drug random effect.

Abbreviations: HR = hazard ratio, OR = odds ratio.

^*^ *p* ≤ 0.1.

**p < 0.05.

***p < 0.01.

In summary, negotiations over public funding exhibit substantial delay, with a median time to agreement of 1.34 years and 71% of negotiation rounds ending without agreement. Higher ICER categories, considerable clinical or economic uncertainty, and high projected budget impact were all associated with significantly lower hazards of agreement. Therapies judged to have clinically important effects, those with FDA priority designation, and those with higher public interest reached agreement more quickly. Within‐therapy analyses show that agency confidence increases and ICER categories decline across negotiation rounds. Sequencing effects for multi‐indication drugs differ by specification, reflecting limited sample size and heterogeneity across indications.

## 7 Discussion

Our findings are consistent with key predictions of dynamic bargaining theory under incomplete information. Therapies with lower expected net value to the agency—proxied by higher ICERs, greater uncertainty, and larger projected budget impact—faced longer delays and lower agreement rates. In contrast, therapies with strong clinical importance, regulatory priority, and elevated public interest were listed more quickly. These results are consistent with a threshold rule in which the agency weighs expected health gains against opportunity costs, subject to feasibility constraints.

A delay in agreement is not merely an administrative lag. Rather, it reflects strategic timing of negotiations: a rational response to evolving evidence, budget constraints, and therapeutic alternatives. Companies may postpone submissions to improve perceived value, while the agency may delay approvals to manage cumulative budget impact or signal caution in the face of uncertainty. Agency confidence in the evidence increased across successive negotiations, consistent with the idea that delay allows information to accumulate and credibility to build. ICER bands declined across rounds, reflecting price concessions over time.

Allowing the baseline hazard to vary across therapeutic classes reveals substantial heterogeneity in delay dynamics. Stratifying on ATC level‐2 captures these therapeutic‐area‐specific continuation values without imposing a common temporal pattern across therapeutic areas with fundamentally different budget pressures and evidence norms. The persistence of the covariate effects under therapeutic area‐specific baseline hazards reinforces that ICER bands, uncertainty, budget thresholds, and public interest influence agreement probabilities in ways consistent with dynamic bargaining, while the therapeutic area‐level heterogeneity reflects the broader portfolio context within which each bilateral negotiation occurs.

Public interest also affects outcomes. Surges in internet searches leading up to decisions were positively associated with agreement, suggesting that heightened interest may increase the perceived urgency or salience of a therapy, rather than simply reflecting promotional activity.

We find limited evidence that therapeutic outside options, measured by potential substitute therapies, materially affect bargaining power or the timing of agreement. In contrast, patient need—proxied by FDA priority review designation—is associated with significantly shorter delays, consistent with the agency accelerating decisions when urgency is high, and substitutes are limited. We also detect a small indirect effect of US prices on the probability of agreement. This pattern is consistent with international reference pricing concerns: higher US prices may raise local bids and ICERs due to spillover risks across markets, potentially influencing the agency's assessment.

Sequencing matters for multi‐indication drugs, but the direction and magnitude depend on specification. Prior listing is positively associated with subsequent agreement in random‐effects models (logit and linear), consistent with reputational screening, although estimates are imprecise. A drug that has already been accepted for one indication may be viewed as clinically reliable, versatile or less risky, shaping expectations or perceived risk and making future submissions for the same drug more likely to succeed. In contrast, the within‐drug models (Cox stratified and the linear fixed‐effects models) indicate a lower rate of agreement for later indications, consistent with reduced marginal contribution when expanding into broader or lower‐value populations (Nash‐in‐Nash logic). Budget concentration risk may also contribute. Once a drug is listed for one indication and generates substantial expenditure, the agency faces greater fiscal and political risk in expanding its coverage. The agency might feel that the most valuable use of the drug has already been listed, or that adding indications stretches the budget too far. As more therapies are listed, the marginal budget impact of further expansions rises, making the agency more cautious about approving additional indications even when clinical benefits remain positive. This is especially likely when the first listing was expensive or backed by strong evidence, making it harder for follow‐on submissions to demonstrate sufficient additional value. Given the limited number of multi‐indication drugs and the heterogeneity across indications, we do not interpret these patterns as evidence for a single dominant mechanism. Instead, the results point to two countervailing forces: informational gains from early success and diminishing marginal value or budget‐concentration risk as coverage broadens.

The supplementary mediation analysis (Appendix 3, Table A.3.4) provides additional insight into potential international spillovers. Australia is a secondary market, and companies may internalize upstream and downstream pricing constraints when formulating bids. We find that higher U.S. prices have a very small indirect effect on the probability of agreement through their influence on the ICER, but no meaningful direct effect on agreement. This suggests that while global pricing constraints shape the offers firms submit, the agency's decision‐making remains anchored in domestic assessments of value rather than external price benchmarks.

The study has limitations. Bargaining positions are shaped by pre‐submission choices that are not observed, including decisions not to submit when international reference pricing constraints make price reductions infeasible. Conditional listings and risk‐sharing arrangements may also influence timing and outcomes but are not directly observable in our data. More generally, the Cox models report conditional associations between time‐varying proxies (ICER band, confidence index, budget indicators, saturation) and the instantaneous probability of agreement. These associations are consistent with the continuation‐value mechanism in our framework, but they are not causal estimates of firms' evidence or pricing decisions. In addition, the empirical variables are imperfect proxies for the underlying structural constructs, raising the possibility of measurement error. For these reasons, we interpret the hazard estimates as evidence consistent with the theoretical framework rather than as structural parameters. The within‐therapy panel analyses help address non‐time‐varying confounding but cannot account for dynamic endogeneity in firms' strategic choices. Finally, because true prices and QALY gains are often confidential, we characterize equilibrium outcomes rather than the distribution of payoffs.

Despite these limitations, the analysis shows that observed patterns of delay and disagreement in public drug funding are consistent with strategic behavior under evolving evidence and portfolio constraints. In this setting, delay can improve decision quality by allowing evidence to mature and by revealing how additional listings affect therapeutic saturation and budget exposure. For companies, the incentives differ: when continuation values are high, accelerating agreement is advantageous, whereas prolonged negotiation can weaken their position. Taken together, the results suggest that the timing of agreement reflects the interaction of strategic behavior, changing information, and the agency's broader portfolio considerations.

## 8 Conclusion

This study links dynamic bargaining theory to empirical patterns in public drug funding, showing that the timing of agreement reflects strategic behavior under evolving evidence and portfolio constraints. By modeling delay as an equilibrium outcome rather than an administrative lag, we highlight how cost‐effectiveness, budget exposure, evidence credibility, and therapeutic saturation shape the path to agreement. The results suggest that delay is not simply passive waiting but part of a strategic process in which both parties weigh the value of waiting against the risks of concession. For the agency, additional time can clarify evidence and reveal how further listings affect expenditure within therapeutic classes; for companies, the incentives to accelerate or defer agreement depend on how continuation values evolve across negotiation rounds and indications. Although the empirical estimates rely on imperfect proxies and cannot identify structural parameters, the observed patterns align with the mechanisms emphasized in dynamic bargaining models. The timing of public subsidy decisions appears to be the outcome of negotiation, not merely the product of a bureaucratic process.

## Funding

A Harris received funding from the Australian Research Council DP 1095691 (https://purl.org/au‐research/grants/arc/DP1095691).

## Conflicts of Interest

J.J. Li and A. Harris have a contract through Monash University to conduct independent evaluations of industry submissions to the PBAC.

## Supporting information


Supplementary Material

## Data Availability

The data used is publicly accessible from https://www.pbs.gov.au/info/industry/listing/elements/pbac‐meetings/psd.

## Endnotes

^1^
There are out of cycle meetings where decisions are made, but these were rare prior to 2019.

^2^
The PBAC may recommend a drug be listed on the PBS pending conditions on coverage. Subsequent confidential negotiations may alter the effective price or eligible population meaning the observed decision does not reflect the final negotiated terms. For this reason, these types of recommendations are coded 0. Note that a recommendation not to list precludes a subsidy on the PBS.
