# Supplementary material for: Strategic Delay and Bargaining Over Public Insurance Coverage for Drugs in Australia
Source: Health Econ. 2026 Mar 12;35(6):1005–18. doi: 10.1002/hec.70095 (PMC13126100; doi:10.1002/hec.70095)
Supplement: Supplementary file 1 — Supplementary Material [file HEC-35-1005-s001.docx]

# **Appendix 1:** **Strategic Foundations and Empirical Estimation of Therapy Listing Decisions**

## Conceptual Framework

We formalise the negotiation process between pharmaceutical companies and the public funding agency using a dynamic Nash-in-Nash bargaining framework embedded within a shared strategic environment. This expands the simplified rule in the main text into its full three-condition form. Each company–agency pair negotiates asynchronously over the listing of a therapy, but the equilibrium outcome reflects interdependencies across therapies, therapeutic classes, and budget constraints.

## Bargaining Structure

Each negotiation is modelled as a sequence of discrete offers from the company. The agency applies a deterministic listing rule: a therapy is accepted if its QALY-equivalent value exceeds its cost, the budget impact is feasible, and it offers incremental value beyond saturation thresholds—that is, it must demonstrate meaningful advantages over therapies already listed in the same drug therapeutic class, especially when the class is crowded, and price premiums are harder to justify. This rule approximates welfare maximisation under institutional constraints and reflects HTA informed drug funding decision making practice in Australia and comparable jurisdictions.

Companies internalise the agency’s evolving portfolio and budget position when choosing when and how to submit. Delay arises not from time discounting (as in (Rubinstein, 1982)), but from strategic anticipation of more favourable listing conditions. This aligns with signalling models (Fudenberg & Tirole, 1986), where delay conveys quality and reshapes expectations.

## Belief Updating and Rule-Based Evaluation

Although the agency applies a structured decision rule, its valuation inputs evolve over time. As new clinical evidence, cost data, and contextual information become available, the agency implicitly updates its beliefs about the value of a therapy. This belief updating is not modelled as Bayesian inference—that is, we do not model Bayesian priors or posterior updates. Instead, we treat the agency’s evaluation as rule-based but dynamically informed. We treat the agency’s evolving inputs as observed time‑varying covariates in the hazard; we do not model the agency’s latent prior/posterior process.

This modelling choice improves tractability in several ways:

- It avoids the need to estimate unobservable priors or simulate belief updates.
- It keeps the focus on company-side dynamics and observable agency thresholds.
- It preserves institutional realism, reflecting how agencies operate in practice.
- It enables modular estimation using standard econometric tools, without requiring dynamic programming or probabilistic inference.

In short, while the agency’s valuation evolves with accumulating evidence, our framework does not treat it as a Bayesian actor. This hybrid structure—strategic company behaviour interacting with evolving but rule-bound agency evaluation—captures the institutional reality of HTA informed decision making and supports empirical identification.

## Strategic Interdependence

The model incorporates strategic interdependence through three channels:

- **Budget constraints**: The agency faces cumulative budget pressure, which influences the marginal value of listing additional therapies. While there is no fixed annual budget, periods of slowed approvals or temporary freezes reflect strategic responses to budget growth.
- **Therapeutic class saturation**: When multiple therapies are already listed within a class, the marginal value of adding another may be reduced due to therapeutic redundancy or budget pressure—a dynamic we refer to as *therapeutic saturation*. Companies may delay submissions to avoid entering a congested class, where the probability of listing is lower and price concessions are more likely.
- **Latent preferences and public pressure**: Companies may invest in marketing or advocacy to influence perceived value and accelerate agreement. These signals interact with saturation and budget dynamics to shape listing outcomes.

## Empirical Mapping

We map the structural model to an empirical hazard framework, where the probability of agreement in each round depends on:

- The expected net benefit to the agency;
- The degree of saturation within the therapeutic class;
- Company outside options and international pricing;
- Marketing investment and public sentiment;
- Temporal dynamics and cumulative budget impact.

The baseline hazard captures the lifecycle of company continuation value, rising as high-value therapies are listed and declining as latent disadvantages accumulate. Saturation is modelled at the therapeutic class level, reflecting localised strategic interdependence.

## Equilibrium Concept

The equilibrium can be interpreted as a dynamic Nash-in-Nash outcome with finite time to resolution. Each bilateral negotiation is resolved optimally, conditional on the strategic environment shaped by other therapies. Companies may delay submission, bundle evidence, or sequence indications to influence agency valuation. The agency may slow approvals in response to cumulative budget pressure or saturation in a therapeutic area.

In this framework, delay is a rational response to strategic interdependence, latent budget dynamics, and the need to signal quality. It reflects the empirical reality of drug funding decision systems, where listing decisions evolve with evidence, portfolio composition, and public engagement—even under a rule-based evaluation framework.

We model the drug listing process as a dynamic strategic interaction between companies and the funding agency. Each company–agency pair engages in bilateral negotiation over the listing of a therapy, embedded within a shared budget-constrained environment. The agency applies a threshold-based rule: therapies are listed if their net health benefit is non-negative, their budget impact is feasible, and their incremental value exceeds a saturation threshold.

This structure aligns with a dynamic Nash-in-Nash bargaining framework, where each bilateral negotiation is resolved optimally, conditional on the strategic environment shaped by other therapies. Although submissions occur asynchronously, companies internalise the agency’s evolving budget position and therapeutic portfolio when choosing their strategies. Both companies and the agency anticipate potential competition: companies may accelerate or delay submissions to avoid drug-class-level saturation or exploit favourable timing, while the agency internalises how cumulative approvals affect budget capacity and the marginal value of listing additional therapies.

When within therapeutic area heterogeneity and saturation are modelled at the therapeutic class level, strategic interdependence is localised within each class. Companies respond to budget saturation and latent preferences specific to their therapeutic category, rather than the entire portfolio. This reflects the empirical reality of HTA systems, where therapeutic context and drug class-specific dynamics shape both company behaviour and agency evaluation.

Importantly, although no fixed budget is allocated in any given period, the agency faces ongoing pressure to maintain the portfolio budget on a sustainable growth trajectory. Periods of slowed approvals or temporary freezes reflect strategic responses to cumulative budget growth, rather than explicit budget caps.

This dynamic Nash-in-Nash formulation reflects the work of Collard-Wexler, Gowrisankaran, and Lee (2019), embedding sequential bilateral negotiations within a shared strategic environment.

## Agency Decision Rule

If all three conditions are met, the therapy is listed $.$

The agency lists therapy *i* at time *t* ($a_{it}$ = 1) only if three conditions are satisfied:

## ****1. Net payoff condition****

$$\psi_{it}-\frac{p_{it}}{\lambda}\geq0.$$

$\psi_{it}$is the agency’s latent valuation of therapy $i$ at time $t$, expressed in QALYs.

$\frac{p_{it}}{\lambda}$is the cost per patient converted into QALY‑equivalent units using the shadow price of a QALY $\lambda$ (assumed time invariant during negotiation)

The valuation term is:

$$\psi_{it}=k\nu_{it}-\rho r_{it}+\gamma\varphi_{it}+\varepsilon_{i},$$

where:

$\nu_{it}$: patient need index

$r_{it}$: evidence risk index

$\varphi_{it}$: public interest index

$k$: QALY reward per unit need

$\rho$: QALY penalty per unit risk

$\gamma$: QALY reward per unit of public interest

$\varepsilon_{i}$: idiosyncratic preference heterogeneity

A therapy satisfies the net payoff condition when its **QALY‑valued benefits exceed its QALY‑equivalent opportunity cost.**

## ****2. Budget feasibility condition****

$$\frac{b_{it}}{\lambda}\leq B_{t}^{resid}$$

$b_{it}$: projected budget impact of therapy *i*.

$\lambda$: shadow price of a QALY, converting budget impact into QALY‑equivalent units.

$B_{t}^{resid}$: residual budget capacity at time *t*.

This prevents the agency from approving therapies that would exhaust or exceed its available resources. It reflects a dynamic budget constraint rather than a fixed cap.

## ****3. Saturation threshold condition****

Let $C\left( i \right)$ denote the ATC class of therapy $i$. Define the number of **other** drugs already listed in that class:

$$n_{-i,t}=\sum_{j\in\mathcal{C}\left( i \right),\text{ }j\neq i} a_{jt}$$

The saturation threshold is:

$$\delta_{it}=\delta\left( n_{-i,t} \right),\delta^{'}\left( \cdot\right)>0$$

As more therapies are listed in a class, the required incremental value rises. This ensures that new therapies offer **meaningful marginal value** in crowded therapeutic areas.

Together, these conditions ensure that therapies are cost‑effective, affordable within the residual budget, and offer incremental value in crowded classes.

## Company Strategy and Continuation Value

Each company chooses a strategy vector:

$$S_{it}=p_{it},\text{ }e_{it},\text{ }\varphi_{it},\text{ }T_{it},$$

where:

- $p_{it}$: proposed price (entering the agency rule as $p_{it}/\lambda$)
- $e_{it}$: evidence package submitted
- $\varphi_{it}$: public interest or visibility signal
- $T_{it}$: timing of submission

The company maximises expected payoff by balancing four components.

1. **Immediate listing payoff**

$$a_{it}\cdot\left( \psi_{it}-\frac{p_{it}}{\lambda} \right)$$

This is the **net QALY‑equivalent payoff** conditional on immediate acceptance.

1. **Evidence generation costs**

$$-c_{i}\left( e_{it} \right)$$

where $C_{i}\left( \cdot\right)$is the cost of producing additional clinical or economic evidence. Higher evidence quality may increase $\psi_{it}$ but reduces net payoff through higher cost.

1. **Public interest payoff**

$$\theta_{i}\varphi_{it},$$

where $\theta_{i}$is the marginal payoff to the company from increased activity to achieve public visibility. Public interest enters the agency’s valuation through $\gamma\varphi_{it}$.

## ****4. Continuation value of delay****

$$V_{i,t+1},$$

the expected future payoff from delaying submission. Delay is optimal when:

$$V_{i,t+1}>\psi_{it}-\frac{p_{it}}{\lambda}.$$

Companies delay when they anticipate:

- lower saturation $\delta_{i,t+1}$
- stronger evidence $e_{i,t+1}$
- improved budget conditions $B_{t+1}^{\text{resid}}$
- more favourable public interest $\varphi_{i,t+1}$

The continuation value satisfies:

$$V_{it}=\mathbb{E}_{t}\left[ a_{i,t+1}\left( \psi_{i,t+1} - \frac{p_{i,t+1}}{\lambda} \right) \right].$$

## Equilibrium Conditions

A dynamic Nash-in-Nash equilibrium arises when:

- Each company–agency pair engages in bilateral negotiation over therapy listing
- The agency applies a threshold rule conditional on budget and valuation
- Companies choose strategies anticipating the agency’s evolving budget and valuation logic
- Negotiations are resolved sequentially, but each company’s strategy is a best response to the strategic environment shaped by other therapies
- Strategic interdependence is localised within drug classes via common agency value

## Structural–Empirical Mapping

We map the structural threshold‑based listing rule into an empirical duration framework using a Cox proportional hazards model with time‑varying covariates and drug area‑level stratification. The hazard of agreement in each period reflects the agency’s evolving assessment of net benefit, budget feasibility, and therapeutic saturation, as well as company‑side strategic factors.

The structural decision rule:

$$\psi_{it}-\frac{p_{it}}{\lambda}\geq0,\frac{b_{it}}{\lambda}\leq B_{t}^{\text{resid}},\psi_{it}\geq\delta\left( n_{-i,t} \right)$$

implies that the probability of agreement depends on observable proxies for:

1. **Valuation components** (need, risk, public interest)
2. **QALY‑equivalent cost**
3. **Budget feasibility**
4. **Therapeutic saturation**
5. **Drug area‑level heterogeneity**

Because the structural cost term $p_{it}/\lambda$ is not directly observed, we use the **ICER** as a monotone empirical proxy for the opportunity‑cost side of the rule. The valuation components enter through their observed indices, scaled in the structural model by $k$, $\rho$, and $\gamma$. Budget feasibility is captured through an indicator of projected expenditure. Saturation is measured by the number of therapies already listed in the same ATC level‑4 class.

The empirical hazard for therapy $i$in therapeutic class $c$at time $t$is:

$h_{\mathrm{it}}\left( t \right)=h_{0,c}\left( t \right)\text{ }\exp\left( \beta_{1}\text{ }\text{ICER}_{it} + \beta_{2}\text{ }\nu_{it} + \beta_{3}\text{ }r_{it} + \beta_{4}\text{ }\varphi_{it} + \beta_{5}\text{ }B_{t}^{\text{resid}} + \beta_{6}\text{ }n_{-i,t} \right)$

where:

- $h_{0,c}\left( t \right)$is an area‑specific baseline hazard (ATC level‑2 stratification)
- coefficients $\beta$correspond to the structural effects implied by the decision rule

This specification allows:

- **time‑varying covariates**, reflecting evolving evidence, pricing, public interest, and saturation at the therapeutic class level
- **drug area‑level heterogeneity**, consistent with strategic interdependence at the broad drug area level
- **nonparametric baseline hazards**, capturing lifecycle dynamics in negotiation

The resulting hazard ratios provide empirical estimates of how each structural component—cost, need, risk, public interest, budget pressure, and saturation—affects the timing of agreement.

# **Table A.1.1. Structural–Empirical Linkage under Threshold‑Based Listing Rule**

| **Empirical Variable** | **Structural Interpretation** | **Structural Notation** | **Predicted Effect on Hazard** |
| --- | --- | --- | --- |
| **ICER** | Proxy for the QALY‑equivalent cost term in the net payoff rule | $\frac{p_{it}}{\lambda}$ | Higher ICER → ↓ Hazard |
| **FDA priority** | Clinical urgency / unmet need | $k\nu_{it}$ | Higher need → ↑ Hazard |
| **Clinical or economic uncertainty** | Evidence risk reducing effective QALY value | $r_{it}$ | Higher uncertainty → ↓ Hazard |
| **Public interest (Google Trends)** | Visibility / advocacy increasing perceived value | $\gamma\varphi_{it}$ | Higher interest → ↑ Hazard |
| **Annual expenditure forecast > $10m** | Budget feasibility (residual capacity less if therapy>$10m) | $B_{t}^{\text{resid}}$ | Less residual budget → ↓ Hazard |
| **Number of drugs in ATC level‑4** | Therapeutic saturation threshold | $\delta\left( n_{-i,t} \right)$ | More saturation → ↓ Hazard |
| **ATC level‑2 strata** | Area‑level heterogeneity in baseline hazard | $\eta_{\text{ATC}\left( i \right)}$ | Varies by area |

Note that continuation values are unobserved but manifest within a therapy in:

• **Repeated submissions:** count of submissions; shorter inter‑submission intervals and systematic evidence upgrades are consistent with positive continuation value.

• **ICER improvements:** within‑therapy downward movement in ICER band across rounds; interpreted as investment to meet agency thresholds.

• **Rising confidence:** index of clinical/economic uncertainty (0–3) increasing across rounds; narrower CIs or more robust sensitivity checks in later submissions.

• **Class‑level saturation:** slowing hazard or lower marginal hazard as ATC‑4 count rises; interpreted as declining marginal continuation value for entrants.

## Conditions for Nash-in-Nash Equilibrium

Our threshold listing rule bargaining framework is consistent with the conditions for a dynamic Nash-in-Nash equilibrium as formalised by Collard-Wexler et al. (2019). In their framework, each bilateral negotiation is resolved optimally, conditional on the strategic environment shaped by other negotiations. The following conditions are met in the HTA therapy listing context:

- **Bilateral Nash Bargaining**: Each company–agency pair engages in a structured negotiation over therapy listing, governed by a threshold rule that reflects latent valuation, budget feasibility, and saturation.
- **Strategic Interdependence**: Companies internalise the agency’s evolving budget position, saturation thresholds, and latent preferences shaped by other therapies. The agency, in turn, anticipates the cumulative impact of approvals on future portfolio capacity.
- **Sequential Resolution**: Submissions occur asynchronously. Each company’s strategy reflects expectations about future budget conditions and rival therapies, while the agency updates its evaluation logic over time.
- **Endogenous Delay**: Companies delay submission when the continuation value of waiting exceeds the immediate payoff. The agency may slow approvals in response to budget pressure or saturation, consistent with dynamic equilibrium behaviour.
- **Marginal Contribution and Comparability**: All value, cost, and budget impact terms are standardised in QALY-equivalent units, ensuring comparability across therapies and satisfying the equilibrium requirement for marginal valuation.

In equilibrium, each company’s strategy is a best response to the strategic environment, and the agency’s listing decisions reflect consistent application of its threshold rule. Although the agency’s objective function is not explicitly specified, its revealed preferences—through $\psi_{it}$and $\delta_{it}$—serve as sufficient conditions for equilibrium behaviour.

Each bilateral surplus depends on therapy‑specific attributes and on aggregate state variables (residual budget and class saturation). Firms’ strategies affect others only through these state variables, satisfying the separability condition required for a Nash‑in‑Nash decomposition (Collard‑Wexler et al., 2019).

# **Appendix 2: Robustness of the Cox model**

**Table A.2.1: Hazard ratios (HR) for covariates on time to agreement from date of first proposal across different model specifications**

|  | **Cox semi-parametric models (HR)** | | | |  | **Parametric proportional hazard models (HR)** | | | **Logit (OR)** |
| --- | --- | --- | --- | --- | --- | --- | --- | --- | --- |
| **VARIABLES** | **primary max 4yr** | **max 4yr**  **no delayed entry** | **all data** | **max 5yr** |  | **Exponential** | **Weibull** | **Gompertz** | **Logit** |
|  | **(1)** | **(2)** | **(3)** | **(4)** |  | **(5)** | **(6)** | **(7)** | **(8)** |
| FDA priority | 1.45** | 1.72*** | 1.46** | 1.48** |  | 1.41** | 1.53** | 1.41** | 1.16 |
|  | (1.03, 2.05) | (1.22, 2.42) | (1.04, 2.04) | (1.05, 2.08) |  | (1.04, 1.93) | (1.08, 2.17) | (1.04, 1.91) | (0.70, 1.92) |
| Therapeutic saturation *(# drugs in ATC level-4 code)* | 0.95 | 1.00 | 0.97 | 0.95 |  | 0.97 | 0.97 | 0.97 | 0.95 |
|  | (0.88, 1.03) | (0.91, 1.09) | (0.89, 1.04) | (0.88, 1.03) |  | (0.89, 1.05) | (0.89, 1.07) | (0.89, 1.05) | (0.85, 1.06) |
| Clinical uncertainty | 0.50*** | 0.53*** | 0.52*** | 0.51*** |  | 0.49*** | 0.46*** | 0.49*** | 0.48*** |
|  | (0.33, 0.75) | (0.35, 0.80) | (0.35, 0.76) | (0.34, 0.76) |  | (0.32, 0.73) | (0.29, 0.72) | (0.33, 0.74) | (0.30, 0.76) |
| Clinically important difference | 1.55** | 1.37* | 1.45** | 1.52** |  | 1.51** | 1.57** | 1.51** | 1.72** |
|  | (1.07, 2.24) | (0.94, 1.99) | (1.03, 2.06) | (1.06, 2.19) |  | (1.05, 2.18) | (1.06, 2.34) | (1.04, 2.17) | (1.05, 2.82) |
| Economic uncertainty | 0.36*** | 0.39*** | 0.35*** | 0.35*** |  | 0.40*** | 0.45*** | 0.40*** | 0.12*** |
|  | (0.26, 0.51) | (0.27, 0.56) | (0.25, 0.50) | (0.25, 0.50) |  | (0.29, 0.56) | (0.31, 0.64) | (0.29, 0.55) | (0.08, 0.19) |
| ICER band (comparisons versus ICER<$15,000) | | | | | | | | | |
| $15,000 - $45,000 | 0.67* | 0.63** | 0.67* | 0.66* |  | 0.70* | 0.61** | 0.70* | 0.84 |
|  | (0.43, 1.04) | (0.41, 0.98) | (0.44, 1.04) | (0.42, 1.02) |  | (0.47, 1.03) | (0.39, 0.96) | (0.48, 1.04) | (0.45, 1.58) |
| $45,000 - $75,000 | 0.55*** | 0.43*** | 0.54*** | 0.55*** |  | 0.57*** | 0.49*** | 0.58*** | 0.41*** |
|  | (0.36, 0.84) | (0.28, 0.68) | (0.35, 0.82) | (0.36, 0.84) |  | (0.39, 0.84) | (0.31, 0.77) | (0.39, 0.85) | (0.21, 0.80) |
| $75,000 – $105,000 | 0.47** | 0.43** | 0.52* | 0.52* |  | 0.48** | 0.43** | 0.49** | 0.40* |
|  | (0.23, 0.95) | (0.21, 0.87) | (0.27, 1.01) | (0.26, 1.01) |  | (0.25, 0.95) | (0.21, 0.87) | (0.25, 0.95) | (0.15, 1.03) |
| $105,000 - $200,000 | 0.22** | 0.21*** | 0.22** | 0.22** |  | 0.22*** | 0.21*** | 0.22*** | 0.11*** |
|  | (0.07, 0.71) | (0.06, 0.66) | (0.07, 0.71) | (0.07, 0.71) |  | (0.07, 0.66) | (0.07, 0.67) | (0.07, 0.66) | (0.03, 0.40) |
| >$200,000 | 0.15*** | 0.11*** | 0.18*** | 0.14*** |  | 0.14*** | 0.12*** | 0.14*** | 0.15** |
|  | (0.04, 0.48) | (0.03, 0.37) | (0.06, 0.52) | (0.04, 0.45) |  | (0.04, 0.45) | (0.03, 0.39) | (0.05, 0.45) | (0.04, 0.65) |
| Budget impact >$10Mil per annum | 0.77 | 0.80 | 0.75* | 0.79 |  | 0.84 | 0.84 | 0.84 | 0.50*** |
|  | (0.57, 1.05) | (0.58, 1.11) | (0.55, 1.01) | (0.58, 1.07) |  | (0.63, 1.13) | (0.60, 1.17) | (0.63, 1.13) | (0.33, 0.75) |
| Google Trends  (each SD above mean) | 1.24*** | 1.26*** | 1.23*** | 1.24*** |  | 1.21*** | 1.26*** | 1.21*** | 1.31*** |
|  | (1.10, 1.39) | (1.11, 1.42) | (1.10, 1.39) | (1.11, 1.40) |  | (1.09, 1.36) | (1.11, 1.42) | (1.08, 1.35) | (1.07, 1.61) |
| ln_p |  |  |  |  |  |  | 1.24*** |  |  |
|  |  |  |  |  |  |  | (1.14, 1.35) |  |  |
| gamma |  |  |  |  |  |  |  | 0.98 |  |
|  |  |  |  |  |  |  |  | (0.84, 1.14) |  |
| Constant |  |  |  |  |  | 1.34 | 1.22 | 1.36 | 4.28*** |
|  |  |  |  |  |  | (0.81, 2.20) | (0.70, 2.12) | (0.82, 2.27) | (1.87, 9.81) |
| Observations | 625 | 629 | 634 | 632 |  | 625 | 625 | 625 | 634 |

ICER=incremental cost-effectiveness ratio (expressed as additional cost per QALY gained), Mil=million, QALY=quality adjusted life year, *** p<0.01, ** p<0.05, * p<0.1; confidence intervals adjusted for therapy clusters. Col 5 shows within drug analysis of the primary dataset (conditioned on strata of the therapeutic class ATC level-2 allowing different hazard rates by drug.

Appendix 3: Extended analysis time to agreement and value to the purchaser

**The evolution of key components of the agency’s valuation within a therapy across successive negotiation rounds: extended analysis**

***Risk as measured by the agency’s confidence in the quality of evidence of cost-effectiveness***

We test if the agency’s confidence in the evidence of cost-effectiveness improves with negotiation across therapies for a drug using (1) a pooled linear regression, (2) a linear regression with drug fixed effects and (3) an ordinal logit regression across rounds of negotiation within a therapy. Confidence is measured as the linear combined index of uncertainty across economic and clinical uncertainty and the belief in clinical effectiveness. The highest index of confidence (3) is no major clinical or economic uncertainty and confidence in a clinically significant effect. The lowest score is 0, indicating uncertainty in each and no confidence in a clinically significant effect of the therapy. The results in Table A.3.1 show a generally consistent pattern across specifications of improved confidence in a therapy in later rounds of negotiation.

**Table A.3.1 Greater confidence in value before and after first therapy agreement and across rounds of negotiation for a therapy: Pooled linear, Linear fixed effect for therapy, and Ordered logit regression of confidence index (0-3; poor-high)**

|  | **Linear (95%CI)** | | **Linear fixed effect (95%CI)** | | **Ordered logit**  **Odds Ratio (95%CI)** | |
| --- | --- | --- | --- | --- | --- | --- |
|  | **(1)** | | **(2)** | | **(3)** | |
| Prior drug agreement (1=yes) | 0.20* | (-0.03, 0.42) | -0.84** | (-1.66, -0.02) | 1.63 | (0.79, 3.34) |
| round=2 | 0.19** | (0.01, 0.37) | 0.42*** | (0.25, 0.58) | 2.29*** | (1.48, 3.55) |
| round=3 | 0.35** | (0.06, 0.65) | 0.82*** | (0.58, 1.07) | 5.95*** | (2.93, 12.07) |
| round>3 | 0.36* | (-0.03, 0.74) | 0.95*** | (0.61, 1.28) | 7.01*** | (3.11, 15.80) |

*** p<0.01, ** p<0.05, * p<0.1; robust standard errors. 634 observations on 400 therapies; controlled for all covariates in the primary duration analysis (Table 3)

***Cost to government***

We test if the total cost to government is more likely to be over the $10m threshold in later bids using a logit, linear fixed effects and ordered logit across therapies for a drug and across rounds of negotiation. Grouping for fixed effect is within the ATC level-2 code for a therapeutic area.

The results in Table A.3.2 show a consistent pattern across specifications of lower projected government expenditure on a therapy after an agreement for the drug and in later rounds of negotiation within therapies. In all specifications, the effects are imprecisely measured with wide confidence intervals.

**Table A.3.2 Probability of the projected annual government spending on a therapy exceeding $10m before and after the first therapy agreement and across rounds of negotiation for a therapy: linear pooled, linear therapy fixed effect and ordered logit**

|  | **Linear**  **(95%CI)** | | **Linear therapy fixed effects (95%CI)** | | **Random therapy effect ordered logit**  **Odds ratio (95%CI)** | |
| --- | --- | --- | --- | --- | --- | --- |
|  | **(1)** | | **(2)** | | **(3)** | |
| Prior drug agreement (1=yes) | -0.05 | (-0.15, 0.05) | -0.05 | (-0.07, 0.18) | 0.81 | (0.53, 1.24) |
| Therapy negotiation |  |  |  |  |  |  |
| round=2 | 0.04 | (-0.05, 0.14) | -0.06 | (-0.14, 0.02) | 1.21 | (0.82, 1.78) |
| round=3 | 0.16** | (0.03, 0.29) | -0.08 | (-0.22, 0.07) | 2.03** | (1.09, 3.79) |
| round>3 | 0.16 | (-0.08, 0.41) | -0.07 | (-0.31, 0.16) | 2.11 | (0.62, 7.14) |

* p<0.10, ** p<0.05, *** p<0.01. 634 observations on 400 therapies within the group variable drug (n=270). N=634. Robust standard errors; controlled for all covariates in the primary duration analysis (Table 3)

*Incremental Cost Effectiveness Ratio*

We test whether the ICER improves across negotiation rounds by modelling the ICER band as a six‑level ordered variable (1 $\leq$$15k/QALY to 6≥$200k/QALY). In the linear fixed‑effects model, we treat this 1–6 scale as approximately continuous to estimate within‑therapy changes over time. Because the ICER bands are ordinal, we also estimate a random‑effects ordered logit model to verify that the qualitative pattern of results does not depend on treating the scale as continuous. The advantage of the linear fixed-effect model is its ability to control for drug-specific unobservable aspects of bargaining power that do not change across rounds, such as a company’s bargaining ability, which may be correlated with the control variables. However, this comes at the expense of efficiency, as it ignores between drug information. Additionally, by assuming continuous outcomes, it risks potential measurement error bias. In the ordered logit regression, we test if the odds of moving to a higher band each round, compared to the odds of being in a lower band, are greater than one, as the number of indications rises, with more rounds of negotiation for a drug, or with a lower U.S. price.

The results are consistent across specifications that ICERs are lower in later negotiation rounds and after the drug has been listed for another therapy (Table A.3.3). Allowing for heterogeneity across therapies improves the magnitude and precision of the estimates.

**Table A.3.3: ICER over time before and after the first therapy agreement and across rounds of negotiation for a therapy fixed effects and random effects linear model of ICER band**

|  | **Linear** | **Linear therapy fixed effects** | **Ordered logit therapy random effects** |
| --- | --- | --- | --- |
|  | **(1)** | **(2)** | **(3)** |
| Prior drug agreement (1=yes) | -0.04 (-0.31, 0.24) | -0.80 (-2.11, 0.51) | 0.88 (0.39, 2.00) |
| Therapy negotiation |  |  |  |
| round=2 | 0.16 (-0.07, 0.39) | -0.22** (-0.43, -0.00) | 0.89 (0.55, 1.42) |
| round=3 | -0.12 (-0.41, 0.16) | -0.55*** (-0.83, -0.26) | 0.46** (0.25, 0.84) |
| round>3 | -0.31 (-0.74, 0.12) | -0.41 (-1.04, 0.22) | 0.47 (0.13, 1.70) |

* p < 0.05, ** p < 0.01, *** p < 0.001. 634 observations on 400 therapies with therapy fixed effects. Robust standard errors.

The estimates of the effect of a unit change in each variable, in (col 1) and (col 2). The ICER band is coded 1–6 and treated as continuous. Ordered‑logit results for odds ratio (col 3) confirm robustness to the ordinal nature of the scale. ICER 6 ≥$200,000; 5=$105,000-$200,000; 4 = 75000-104,000; 3=$45,000-$74,000; 2= 15,000-44,000 to 1=<$15,000 per QALY; controlled for all covariates in the primary duration analysis (Table 3)

These within‑therapy patterns are consistent with strategic evidence investment but do not by themselves rule out reverse causality or time‑varying confounding. Therapies reaching later rounds are a selected subset; within‑therapy fixed effects reduce but do not eliminate bias from time‑varying selection.

Effect of a prior drug agreement on later therapy agreement

The estimated effects of the prior listing of the drug on the probability of recommending a subsequent therapy were presented in Table 6.

In the logit and linear random effects models, the coefficient on prior accepted drug is positive, indicating that therapies submitted after a prior listing for the same drug are more likely to be accepted. They both have wide confidence intervals. Drugs with an earlier accepted indication tend to have higher overall acceptance rates. This pattern may arise from a drug that has already been accepted for one indication and may be viewed as clinically reliable, versatile or less risky, shaping expectations or perceived risk and making future submissions for the same drug more likely to succeed. However, fixed effects models yield a negative coefficient, suggesting that, conditional on the drug, subsequent submissions are less likely to be accepted. This within-drug effect suggests that once a drug has already been accepted for one indication/condition, later submissions for the same drug may face tougher scrutiny. The agency might feel that the most valuable use of the drug has already been listed, or that adding indications stretches the budget too far. This is especially likely when the first listing was expensive or backed by strong evidence, making it harder for follow-on submissions to demonstrate sufficient additional value.

For multi-therapy drugs, the data do not offer a precise estimate of the direction or magnitude of the effect of prior listing on the probability of subsequent agreement for later therapies, conditional on the predictors in our model. We cannot be confident that any effect exists. This is partly due to sample limitations: only 57% of drugs have more than one therapy, and those with multiple therapies often exhibit substantial heterogeneity. Some drugs follow a clear therapeutic sequence within a disease, while others extend into adjacent disease areas—such as across cancer stages or types. In other cases, therapies target entirely distinct patient populations. These patterns may reflect strategic design but often arise through incidental discovery. As a result, while strategic sequencing in company behaviour is plausible, it is difficult to detect in this dataset, given the noisy and uneven nature of therapeutic development and application.

**Spillover effects**

Australia is a secondary market, with most drugs launched in the United States and Europe before Australia, with several downstream countries after that. Consequently, the price and value offered may take account of the upstream and downstream implications for future pricing for the company. We expect that for some drugs, the US price indirectly impacts the probability of an agreement through its effect on a threshold price and, consequently, the ICER in the bid. There may be some direct effect on the company’s bargaining power if a low overseas price makes the agency unwilling to accept a high ICER. It is not clear that the agency looks to overseas prices in this way, so we do not expect a large direct effect on the time to listing, which is why we exclude the international price from the duration analysis in the main analysis. We use a mediation analysis of the US price to examine the direct and indirect effects of the US therapy price on the probability of an agreement at each negotiation point.

We used a generalised structural equation model (GSEM) to estimate the direct, indirect, and total effects of the log-transformed price of the therapy on the probability of an agreement mediated by the ordinal variable representing ICER bands. The model is specified as follows:

1. **Mediator Equation (Ordinal Outcome):**

ICER_i_=α_0_+α_1_⋅(ln)USprice_i_ +ε_1i_,

where ICER is treated as an ordinal variable with categories determined by latent thresholds. The equation was estimated using an ordinal logistic regression

1. **Outcome Equation (Binary Outcome):**

logit(Pr(agreement=1))=β_0_+β_1_⋅ln(USprice_i_)+β_2_⋅ICER+β3⋅CinicalUncertainty+β4⋅Clinical Importance +β_5_⋅EconomicUncertainty +β_6_⋅GoogleTrends +β_7_⋅Substitutes+β_8_⋅BudgetCost+ε_2i_.

**Table A.3.4 The direct effect of US price(log) on the probability of an agreement (logit) and the effect mediated through the ICER (ordered logit)**

**Panel 1: US price(log) mediation analysis GSEM**

| **ICER** | **Coefficient (95%CI)** |
| --- | --- |
| US Price(log) | 0.08*** (0.05, 0.11) |
| **Agreement (yes=1)** |  |
| ICER | -0.49*** (-0.69, -0.29) |
| US Price(log) | 0.04 (-0.02, 0.09) |
| Clinical Uncertainty | -0.74*** (-1.21, -0.28) |
| Clinical effectiveness | -0.43* (-0.88, 0.02) |
| Economic Uncertainty | -2.06*** (-2.50, -1.63) |
| Google trends (each SD above mean) | 0.28***(0.07, 0.49) |
| FDA priority | 0.08 (-0.41, 0.57) |
| Therapeutic saturation (# drugs in ATC level-4 code) | -0.06 (-0.17, 0.06) |
| Budget impact >$10million per annum | -0.69*** (-1.12, -0.25) |
| Constant | 2.40*** (1.64, 3.15) |

**Panel 2: Direct and Indirect Effect of a change in US price of $5000 from mean of $9210 on Agreement**

|  | **Change in probability of an agreement** |
| --- | --- |
| Indirect | -0.005*** (-0.007, -0.002) |
| Direct | 0.004 (-0.0003, 0.009) |
| Total | 0.0003 (-0.005, 0.005) |

* p < 0.05, ** p < 0.01, *** p < 0.001. n=634

The total effect of the US price on the probability of an agreement is the combination of the direct and indirect pathways. Table A.3.4 shows that the indirect effect, while small, is statistically significant, suggesting that the ICER plays a role in mediating the small effect of a higher US price on the probability of an agreement. After accounting for this mediation, the direct relationship between price and agreement is weak and not statistically significant. In summary, higher US drug prices indirectly decrease the probability of recommending the drug through their influence on reducing the agency’s value of the therapy (moving into less favourable ICER categories), but this effect is very small. For example, an increase in the US price of $5000 from the mean price of $9210 would result in a fall in the probability of agreement of 0.005 points, if not counteracted by a positive direct effect on agreement or confounded by any unobserved characteristics of the drug that are correlated with the US price but not with the ICER.

Appendix 4: test for the proportionality assumption

**Table A.4.1: Post estimation test for the proportionality assumption**

|  | **rho** | **chi2** | **df** | **Prob>chi2** |
| --- | --- | --- | --- | --- |
| FDA priority need | -0.02095 | 0.09 | 1 | 0.7658 |
| Saturation | 0.06454 | 0.74 | 1 | 0.3911 |
| Clinical uncertainty | -0.04644 | 0.55 | 1 | 0.4595 |
| Clinically important difference | 0.05153 | 0.6 | 1 | 0.4399 |
| Economic uncertainty | 0.03395 | 0.29 | 1 | 0.5884 |
| ICER category (comparisons versus ICER<$15,000) |  |  |  |  |
| $15,000 - $45,000 | -0.04501 | 0.39 | 1 | 0.5311 |
| $45,000 - $75,000 | 0.028 | 0.13 | 1 | 0.7214 |
| $75,000 - $105,000 | 0.10373 | 1.77 | 1 | 0.183 |
| $105,000 - $200,000 | 0.02893 | 0.15 | 1 | 0.6962 |
| $200, 000 | -0.0187 | 0.07 | 1 | 0.7848 |
| Budget impact >$10million per year | 0.09863 | 1.65 | 1 | 0.1989 |
| Google Trends (each SD from mean) | -0.0799 | 1.31 | 1 | 0.2524 |
| Global test |  | 8.97 | 12 | 0.7056 |

Table Note: Tests of the proportional-hazards assumption using Schoenfeld residuals. Robust variance–covariance matrix used.

# **Appendix References**

Collard-Wexler, A., Gowrisankaran, G., & Lee, R. S. (2019). “Nash-in-Nash” bargaining: A microfoundation for applied work. *Journal of Political Economy, 127*(1), 163-195.

Fudenberg, D., & Tirole, J. (1986). A theory of exit in duopoly. *Econometrica: Journal of the Econometric Society*, 943-960.

Pekarsky, B. A. K. (2015). *The New Drug Reimbursement Game*

*A Regulator’s Guide to Playing and Winning*. Switzerland:

Springer International Publishing.

Rubinstein, A. (1982). Perfect equilibrium in a bargaining model. *Econometrica: Journal of the Econometric Society*, 97-109.
